# Chromatin insulators in gene regulation and 3D genome organization

**DOI:** 10.1042/BST20253036

**Published:** 2025-10-28

**Authors:** Hina Sultana, Rohit Kunar, A. Gregory Matera

**Affiliations:** 1 Integrative Program for Biological and Genome Sciences, University of North Carolina, Chapel Hill, NC, U.S.A.; 2 Department of Biology, University of North Carolina, Chapel Hill, NC, U.S.A.; 3 Department of Genetics, University of North Carolina, Chapel Hill, NC, U.S.A.; 4 RNA Discovery and Lineberger Comprehensive Cancer Center, University of North Carolina, Chapel Hill, NC, U.S.A.

**Keywords:** chromatin, DNA binding, epigenetics, epigenomics, gene expression and regulation, transcription

## Abstract

The human genome attains an amazing spatial organization in the packaging of 2 m of DNA into a 10-μm nucleus. Such structural organization is achieved by the folding of chromatin and the regulation exerted by architectural proteins such as insulators. Chromatin insulators are boundary elements of the genome that, through enhancing blocking activities, demarcation of chromatin domains, and chromatin looping, regulate transcription. The review focuses on the identification and characterization of insulators in various species, discussing mainly the functions of the CCCTC-binding factor (CTCF) in mammals and functionally equivalent insulator proteins in *Drosophila melanogaster*. We review here the mechanisms of enhancer blocking, barrier activity, and loop extrusion, emphasizing their effects on topologically associating domains and chromatin architecture. Furthermore, we discuss new concepts that have come into prominence: tethering elements and redundancy among the insulator proteins, which contribute to chromatin organization. Advances in methodology, including chromosome conformation capture and high-resolution imaging techniques, have transformed our view of the dynamic interplay between the architecture of chromatin and transcription regulation. This review discusses the importance of insulators for genome organization and describes future directions in investigating their roles in both gene regulation and three-dimensional genomic architecture.

## Introduction

The human diploid genome, consisting of ∼6.2 billion DNA base pairs, is roughly 2 m in length and must be packaged into a nucleus merely 10 μm in diameter. This remarkable feat of packaging begins with wrapping the DNA around histones to form chromatin. Approximately 147 base pairs of DNA are wrapped around an octamer of four histone subunits (H2A, H2B, H3, and H4) to form a nucleosome core particle [1]. The chromatin then folds upon itself to form higher order structures [2,3]. Post-translational modifications of histones, such as acetylation, methylation, phosphorylation, and ubiquitination, regulate chromatin structure and function [4]. Histones thus provide structural stability to chromatin while controlling accessibility to regulatory factors [5]. The dynamic process of genome unfolding and refolding during cycles of transcription and replication has been a subject of intense research over the past few decades. Architectural proteins such as insulators play a fundamental role in this genomic organization [6]. This review will explore the role of insulator proteins in transcriptional regulation and genome function.

## Discovery and characterization of chromatin insulators

The concept of chromatin insulators emerged in the mid-20th century from studies on *Drosophila melanogaster*. Researchers discovered DNA elements capable of blocking enhancer–promoter (E–P) communication, leading to the identification of specialized insulator sequences. These sequences act as genomic boundaries, organizing the genome into distinct functional domains and regulating gene expression. One of the earliest identified insulators was the specialized chromatin structure (scs) and its counterpart scs’, discovered near *Drosophila* heat-shock gene [7–10]. Other notable *Drosophila* insulators include Frontabdominal-7 (Fab-7), Frontabdominal-8 (Fab-8), and Mcp within the bithorax complex [11–17]. The first vertebrate insulator, the HS4 element, was found at the 5′ end of the chicken β-globin locus [18]. In mammals, the *H19* imprinting control region (ICR), which regulates *H19* and *I*expression, is another well-studied insulator [19].

## Insulator binding proteins

Insulators function through binding with specific proteins, termed insulator binding proteins (IBPs). Several DNA-binding proteins have been implicated in insulator function, with each protein contributing unique architectural and regulatory properties to chromatin organization. Among these, CCCTC-binding factor (CTCF) stands out as a central player in vertebrate insulator function. CTCF is a highly conserved, multifunctional zinc finger DNA-binding protein that plays a central role in genome organization and transcriptional regulation. Structurally, CTCF harbors 11 zinc finger domains that enable versatile and context-specific binding to DNA. Although initially it was thought that CTCF binds to the CCCTC motif, later genome-wide studies showed that CCCTC is not the canonical motif and CTCF preferentially binds to a longer, more complex site [20–25]. In *D. melanogaster,* several IBPs have been identified. Boundary element-associated factor (BEAF)-32 contains zinc finger domains that confer its ability to bind to a palindromic DNA sequence [11]. BEAF binds to thousands of regions in the *Drosophila* genome, but not all of them function as insulators. Suppressor of Hairy-wing [Su(Hw)] and centrosomal protein 190 (CP190) are other IBPs that contribute to insulator activity by mediating long-range interactions and preventing inappropriate E–P communication [26–31].

## Models of transcription regulation by chromatin insulators

### Enhancer blockers

**Figure 1:**
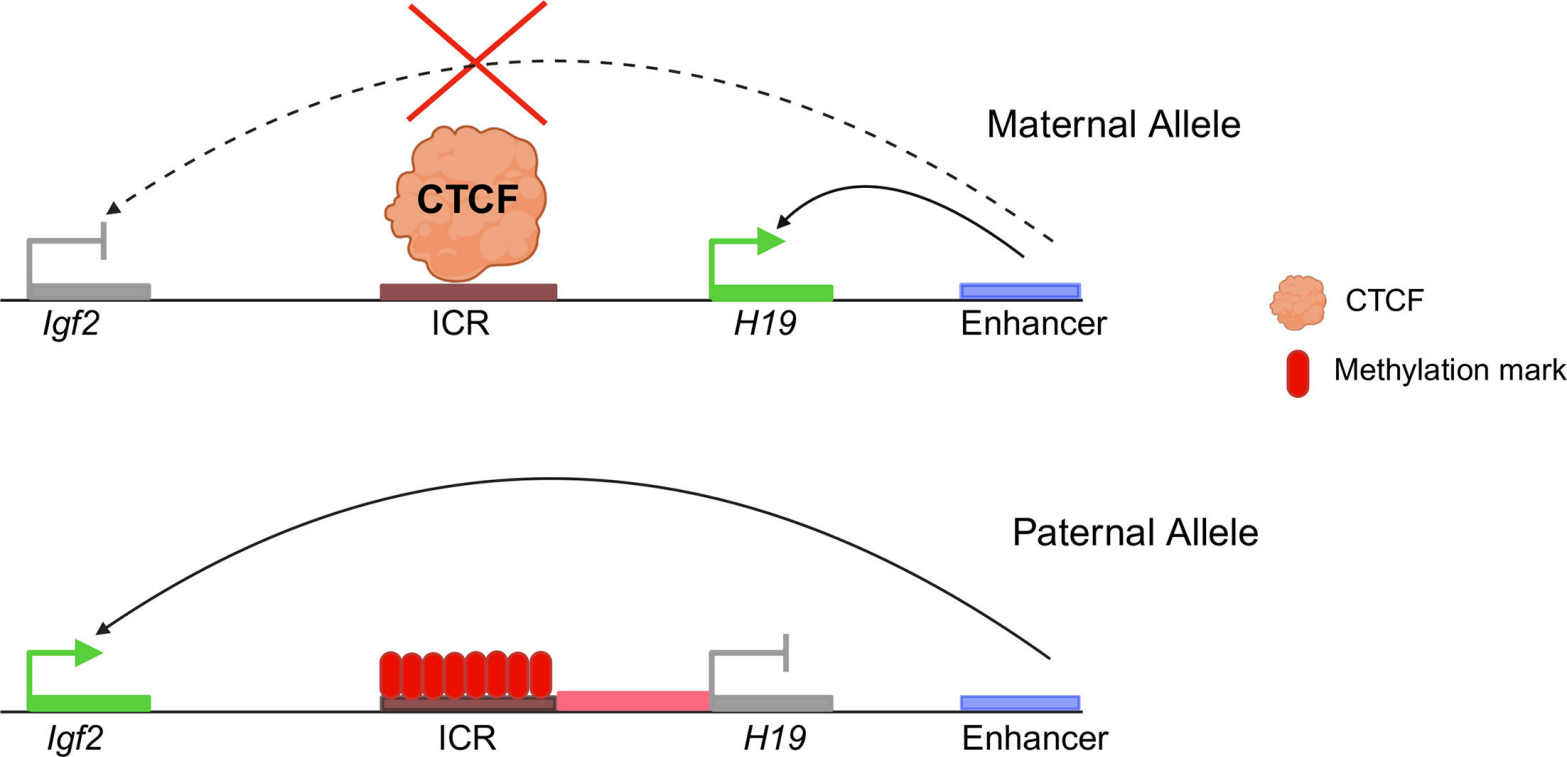
The *Igf2/H19* imprinting control region ( ICR) of mouse. The ICR (brown rectangle) contains CTCF binding sites. On the maternal allele, CTCF binds the ICR to insulate *Igf2* from the shared enhancer; the dotted arrow with a cross (×) denotes the blocked enhancer–*Igf2* contact, resulting in *Igf2* repression. On the paternal allele, DNA methylation (red ovals) prevents CTCF binding, allowing the enhancer to activate *Igf2* while *H19* is silenced. Schematic; not to scale. ICR—brown rectangle; methylation marks—red ovals; CTCF—orange sphere; enhancers—purple rectangles; active promoters—green arrows; inactive promoters—gray vertical bars; blocked enhancer–promoter contact—dotted arrow with ×. Adapted from Iqbal H, Mishra R. Chromatin Domain Boundaries: Defining the functional Domains in Genome. Proc Indian Natn Sci Acad. 2007, 73: 239-253.

Enhancers are *cis*-regulatory elements that modulate gene expression by binding transcription factors and recruiting the transcriptional machinery to target gene promoters. According to the enhancer blocker model, an insulator, when present between an enhancer and a promoter, will not allow the enhancer to act on the promoter. However, the enhancer can act on the promoter when the insulator is present at any other location [32]. Some of the most well-studied enhancer blocker insulator elements are present at the Hox locus in *D. melanogaster*. Fab-7, Fab-8, and Mcp insulator elements are essential for spatiotemporal regulation of *hox* genes [11]. The ME insulator element is present between the *myoglianin* and *eyeless* genes in *Drosophila melanogaster*, and it shows enhancer blocking activity in transgenic assays [33]. CTCF was identified in mammals as an IBP, and initial studies described CTCF as an enhancer blocker [34]. At the *Igf2-H19* imprinted domain in mice, *Igf2* is only expressed from the paternally derived allele, whereas *H19* is only expressed from the maternal allele. Between the two genes is a region known as the *H19* ICR, where the paternal allele is methylated. The CTCF protein interacts with an insulator element present in the ICR. Since the ICR in the maternal allele is not methylated, CTCF binds to it, causing it to operate as an insulator and preventing downstream enhancers from acting on the *Igf2* promoter. When it comes to paternal alleles, the methylation of the ICR prevents CTCF from binding to it, which results in the loss of insulator function. It is now possible for the downstream enhancer to activate *Igf2* [19,35] (Figure 1).

### Barriers

In addition to facilitating enhancer–promoter communication, chromatin insulators also serve as boundary elements that demarcate functional genomic domains and prevent the spread of repressive chromatin marks into active regions or vice versa. *scs* and *scs’* elements flank the *hsp70* genes in *D. melanogaster*. On giving heat shock, this region becomes transcriptionally active, but the active domain is contained between the scs and scs’ elements, suggesting that they act as boundaries of the heat shock locus [7–10]. These elements prevent the active chromatin from entering the nearby regions working as barrier elements. Both scs and scs’ were also shown to possess enhancer blocker activities in transgenic assays. BEAF binding is shown to be necessary for the barrier function of scs’ [7,36]. Zeste-white 5 (Zw5) binds to the scs element and confers its insulator function [37,38]. The chicken β-globin locus that contains four globin genes has several DNase I hypersensitive sites present both upstream and downstream of this locus. The 5′HS4 site present upstream of the locus was shown to block the spread of heterochromatin in the locus. The 5′ HS4 element was also shown to possess insulator blocker activity in transgenic constructs. The enhancer blocker function of 5′HS4 is attributed to the CTCF protein, whereas the barrier function is attributed to upstream stimulatory factor 1 [18,23,34,39–41]. More recently, Nora et al. used an auxin-inducible degron system to acutely deplete CTCF in mouse embryonic stem cells. This resulted in the erasure of boundaries but did not lead to the spreading of H3K27me3 into active regions [42].

## Topologically associating domains

**Figure 2:**
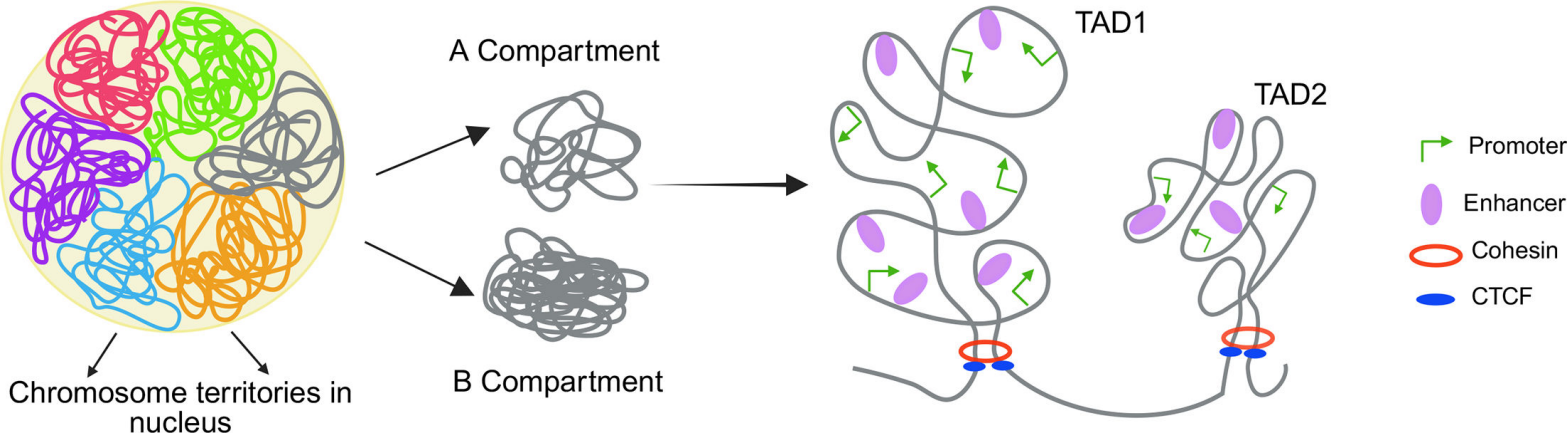
Three-dimensional genome organization. Chromosomes occupy specific regions in the genome termed chromosome territories which are further compartmentalized into A and B compartments. A compartments are enriched in open, gene-dense, transcriptionally active chromatin, whereas B compartments are enriched in more compact/heterochromatic, gene-poor chromatin. Within compartments, topologically associating domains (TADs) form self-interacting units that are separated by insulator elements. Enhancer–promoter (E–P) communication is many-to-many: individual enhancers can contact multiple promoters, and single promoters can integrate inputs from multiple enhancers. Contacts are typically more frequent within a TAD than across TAD boundaries. The schematic shows two mammalian TADs separated by CTCF and cohesin. Key: promoters—green arrows; enhancers—purple spheres; cohesin—red rings; CTCF—blue ovals. Created in BioRender. Sultana, H. (2025) https://BioRender.com/fnm55mz.

One of the hallmark functions of insulator elements is their ability to mediate long-range chromatin interactions, bringing distal regulatory elements, such as enhancers and silencers, into spatial proximity with their target genes. By forming chromatin loops, insulators facilitate the establishment of regulatory contacts essential for gene activation or repression. Topologically associating domains (TADs) are chromatin units that show high interaction within themselves but are insulated from other TADs by insulator proteins. Even though the two DNA sections are proximal to one another in the linear genome, chromatin interactions within TADs are more common than those between TADs (Figure 2). E–P loops are preferably localized to insulated TADs, thereby regulating gene expression.

### Mammalian TADs

In mammals, TADs are produced by chromatin extrusion through cohesin. Loop extrusion was earlier described to be bidirectional [43], but a recent study suggests that the extrusion is unidirectional with frequent orientation switching. Extrusion is halted when cohesin encounters a convergently oriented CTCF [44] (Figure 2). This orientation of CTCF is critical in stopping the loop extrusion [45,46]. Strong TAD boundaries have multiple CTCF-binding sites [47–50]. Loop extrusion is a dynamic process, as cohesin complex keeps associating and dissociating from chromatin. CTCF binds to DNA for an average of 1–2 min, whereas cohesin binds to DNA for 10–20 min [51]. Upon CTCF dissociation, loop extrusion can continue, resulting in larger loops. *In vitro* studies using non-chromatinized DNA have shown that DNA tension plays a role in modulating the permeability of chromatin loops [52]. While CTCF bypass may happen at low tension, it prevents loop extrusion at high tension. Also, at high tension, backtracking of the loop can occur. These studies suggest that CTCF may act as an active regulator of loop extrusion [52,53]. Loss of CTCF in mouse embryonic stem cells resulted in a loss of TAD organization with only modest effects on gene regulation [42]. In a study done in mice, Lupiáñez et al. engineered structural variants that disrupted the TAD boundary between *Epha4* enhancers and neighboring genes such as *Wnt6*, *Ihh*, and *Pax3*. This led to ectopic activation of these normally silent genes in limb tissue, resulting in various limb malformations [54]. Building on this concept, a more recent study performed by Anania et al. dissected the same locus at a higher resolution by deleting individual and clustered CTCF binding sites at the *Epha4–Pax3* boundary. These deletions weakened boundary insulation, caused ectopic activation of the *Pax3* gene in developing limbs, and resulted in graded brachydactyly phenotypes in mice [47]. Acute depletion of CTCF using a degron system results in abolishment of TAD borders but does not have significant influence on gene expression. However, a subset of genes is deregulated, likely reflecting the fact that many genes do not rely on long-range enhancers and that enhancer usage is often cell-type specific [55]. Taken together, acute architectural perturbations such as rapid CTCF or cohesin depletion usually weaken TAD insulation. Most steady-state transcription changes little under these conditions, consistent with enhancer redundancy and some promoter autonomy. By contrast, locus-specific boundary loss or disease-driven erosion can permit enhancer hijacking and produce clear transcriptional and developmental effects. Thus, TADs act mainly as organizational scaffolds: often dispensable at homeostasis yet decisive in particular regulatory geometries and during development or pathology [42,47,54–56].

Cohesin is a multiprotein complex. Knockdown of cohesin components also leads to disruption of TADs. Elimination of cohesin components RAD21/SCC1 or its loading factor nipped-B-like (NIPBL) results in elimination of all loop domain TADs [57,58]. Wings apart-like protein homolog (WAPL) is required for the release of cohesin from DNA. Removal of WAPL leads to stronger binding of cohesin to chromatin resulting in stronger TADs [59,60]. Cohesin contains an ATPase domain SMC3. Mutation of *SMC3* results in loss of TADs, suggesting that loop extrusion is an active process [61]. Loss of CTCF resulted in loss of borders at TADs, but overall compaction was not affected by the loss of CTCF, but the loss of cohesin resulted in an overall loss of compaction [42,58]. TADs are transient, and the actual position of a TAD border may not be fixed but change with space and time. A recent study found that TADs are conserved between young (2 month) and old (28 months) mouse skeletal muscle stem cells [62]. Myc-associated zinc finger protein (MAZ) has been shown to have an accessory role in loop extrusion. MAZ interacts directly with cohesin subunit RAD21. Cohesin relocates to MAZ-binding sites when CTCF is depleted. Deletion of MAZ did not result in significant changes in genome organization [63,64]. Large proteins such as RNAPolII may also interfere with loop extrusion [65].

#### *Drosophila melanogaster* TADs

In *Drosophila,* evidence of loop extrusion through cohesin is lacking, suggesting the presence of alternate mechanisms for looping. Also, *Drosophila* CTCF is not enriched at TAD borders; rather, other insulator proteins such as BEAF, CP190, and Chro are found at TAD borders [66–71]. These insulator complexes interact with themselves and facilitate chromatin looping. It was found that BEAF is enriched at TAD borders in Kc167 cells, but knockdown of BEAF in these cells had a negligible effect on three-dimensional (3D) chromatin organization [67]. A plausible explanation for this could be that BEAF shares a similar binding motif with DNA replication-related element-binding factor (DREF) [72,73]. Therefore, it is possible that in BEAF knockdown cells, DREF occupies these motifs. Depletion of BEAF in BG3 cells leads to partial loss of TAD borders, but double knockdown of BEAF-32 and DREF lead to a significant loss of TAD borders. Double knockdown of CP190 and Chro in BG3 cells also resulted in a loss of TAD borders [74]. Knockdown of BEAF in BG3 cells resulted in changes in genome architecture, along with changes in gene expression. Upon BEAF knockdown, it was found that TADs are reorganized and TAD borders found in heterochromatic regions are lost. The borders not affected by depletion of BEAF, Cp190, and Chro were found to be enriched for CTCF, cohesin, Mediator Complex components, and Trithorax group proteins [74]. Different insulator proteins are present at different TAD borders, and depletion of a subset of these proteins resulted in weakening of those borders where they were present, with mild or no effect on other borders. Treatment of Kc167 cells or *Drosophila* embryos with transcriptional inhibitors resulted in more inter-TAD interactions than intra-TAD interactions [75,76]. Strongest TAD borders are associated with highly transcribing genes [77]. In *Drosophila* at the *bitesize* locus, the promoter is located next to a CP190 binding site at a TAD boundary. Deletion of the promoter results in a greater loss of TAD boundary than the deletion of the CP190 binding site [78]. Whereas most architectural proteins are enriched in all E–P loops, housekeeping genes are more likely to have CP190 and BEAF-32 sites, whereas developmental genes are more likely to have Fs(1)h and Rad21 sites [70]. Remarkably, only about 8% of the domain borders in *Drosophila* larvae include CTCF, the most abundant boundary element in mammalian cells. Thus, CTCF depletion has minimal impact on the overall chromatin structure [79]. Fub and Fab-7 insulator elements present in the bithorax complex of *D. melanogaster* have binding sites for CTCF and Rad21. Loss of these elements results in numerous erroneous E–P connections due to merging of the two nearby TADs. This leads to ectopic gene expression and developmental abnormalities [80]. Loss of any one insulator protein does not result in loss of TAD boundaries due to redundancy in insulator proteins. A recent study further emphasizes the importance of boundary elements in restricting Polycomb domains and ensuring proper promoter regulation. Deletion of the Fab-7 insulator was shown to cause cell type–specific fusion of neighboring domains. This led to the spreading of H3K27me3 and deregulation of Abdominal-B (Abd-B) promoters [81]. More recently, Denaud et al. demonstrated that physical chromatin domains shape the size and formation of H3K27me3 domains, rather than being established by them. They deleted and relocated the Polycomb response element (PRE) at the *dachshund* locus and found that TAD boundaries limit Polycomb domain spreading and constrain long-range looping between PREs. This study highlights a general role for chromatin architecture in modulating epigenetic regulation [82].

#### Comparative principles: mammals vs. *Drosophila*

In mammals, high-resolution Hi-C studies have identified about 10,000 chromatin loops and many contact domains, with a typical size of ~185 kb [83]. On Hi-C maps, these domains show up as squares along the diagonal, and many of them are bordered by loops that appear as sharp ‘corner dots,’ reflecting strong interactions between the edges of the domain. In *Drosophila*, however, loop extrusion has not been demonstrated, and Hi-C maps lack the corner dots seen in mammals. Instead, the fly genome is organized into physical domains that show more uniform contact patterns [84]. The boundaries of these domains often align with regions marked by distinct histone modifications, such as active (H3K4me3) or Polycomb-repressed (H3K27me3) chromatin [84]. Insulator proteins, including CP190, BEAF-32, and CTCF, are also enriched at these domain boundaries. In mammals, histone modification patterns are most clearly linked to the smaller contact domains, while larger loop structures are mainly shaped by CTCF–cohesin interactions rather than chromatin state [83]. Together, these studies reveal a key difference in genome organization: mammalian chromatin folding is strongly influenced by loop extrusion and loop domains, whereas in *Drosophila,* the domain structure largely mirrors the underlying chromatin landscape.

Studies over the past decade show that the way TADs are formed is not the same in all organisms. In *Drosophila*, polymer modeling studies have suggested that chromatin folding can be explained largely by the epigenomic landscape. Domains with similar chromatin states tend to cluster together, producing TAD-like structures [85]. Later work in mammals showed a different principle. When CTCF was rapidly depleted using degron systems, TAD boundaries were lost, while the broader A/B compartments were maintained [42]. These findings indicate that, unlike in flies, mammalian TADs depend strongly on boundary elements bound by CTCF, whereas chromatin states correlate more strongly with A/B compartmentalization.

## Tethering Elements

Very recently, a new class of regulatory DNA elements was identified, termed as tethering elements (TEs). TEs bring distal regions of the genome together by looping. Each tether element is around 500 bp in length and contributes to soft borders within TADs compared with the hard borders of insulators. Micro-C data show that there are approximately 500 TEs active in *Drosophila* embryos [86,87]. Deletion of TEs eliminates focal contacts and delays activation without perturbing surrounding TADs, indicating that tethers and insulators act via independent mechanisms. Additionally, TEs are said to be distinct from insulator elements as they lack CTCF-binding sites but have binding sites for GAGA-associated factor (GAF) [88]. TEs can work over very large distances ranging up to several megabases to bring regions of the genome together. Nevertheless, only approximately 7% of all tether–tether regulatory loops are disrupted by mutations in GAF, suggesting that additional unknown components are also essential for these high-frequency interactions [88].

In mammals, elements with similar properties have been described as ‘facilitators’ within multipartite super-enhancers (SEs). At the mouse α-globin SE, facilitators lack intrinsic enhancer activity yet are required to potentiate classical enhancers. Moreover, when the human β-globin locus control region (LCR) HS1 element, which by itself has no enhancer activity, was inserted into the mouse α-globin SE at the position normally occupied by a facilitator, it was able to function in this role. This experiment demonstrates that facilitator activity is not sequence-specific but depends on genomic context and position, raising the possibility that such elements could be a more general feature of vertebrate SEs [89]. Tethers in flies and facilitators in mice can be considered conceptually related classes of regulatory DNA that do not act as enhancers per se but, instead, potentiate enhancer function and organize regulatory communication at different scales, tethers by stabilizing distal focal contacts and facilitators by acting within SEs.

## Insulator dysfunction in human disease

Human isocitrate dehydrogenase (IDH) mutant gliomas are associated with DNA hypermethylation. Because of this hypermethylation, CTCF binding is disrupted at a *platelet-derived growth factor receptor alpha* (*PDGFRA*) TAD border. This leads to ectopic activation of the PDGFRA oncogene [56]. In patient-derived IDH-mutant glioma models, pharmacologic demethylation restored CTCF occupancy at this boundary and attenuated *PDGFRA* expression, indicating that boundary erosion is, in part, reversible [56]. The *IGF2-H19* region is controlled by an imprinting center where CTCF acts as an insulator, and its binding depends on DNA methylation. In Beckwith-Wiedemann syndrome, which causes prenatal overgrowth and raises cancer risk, the imprinting center becomes too heavily methylated. This prevents CTCF from binding to the maternal chromosome, the insulation breaks down, and *IGF2* is switched on from both copies. In Silver–Russell syndrome, which is linked to poor growth before birth, the imprinting center is not methylated enough. Here, CTCF binds to both chromosomes, blocking enhancer access, which lowers *IGF2* activity and increases *H19* expression [90]. Cornelia de Lange syndrome (CdLS) provides another example of how disrupted boundary function contributes to human disease. Patient cells carrying *NIPBL* mutations show defective cohesin redistribution, with cohesin accumulating abnormally at CpG islands while being depleted from CTCF-bound sites. This impairs loop extrusion, weakens intra-TAD contacts, and misregulates key developmental genes such as HOX clusters and protocadherins, consistent with the skeletal and neurodevelopmental features of CdLS [91].

## Recent technological advances in the study of chromatin architecture

Our capacity to study chromatin architecture and long-range interactions has been transformed by chromosome conformation capture (3C) and its offshoots, such as 4C, 5C, and Hi-C [92–95]. [87]. However, all 3C-derived maps share core constraints; they are fixed-cell, crosslink- and ligation-based population averages with distance-dependent decay and fragmentation/coverage biases. These techniques report contact frequency, not physical distance or causality [96–98]. Building on these foundations, next-generation approaches address parts of these limitations in complementary ways. Single-cell Hi-C, including combinatorial-indexed sci-Hi-C and single-nucleus Hi-C, adapts proximity-ligation to individual cells, revealing cell-to-cell heterogeneity in 3D genome folding and cell-state differences albeit with characteristically sparse per-cell contact matrices [99–101]. Capture-C [102] and Capture Hi-C [103] concentrate sequencing at chosen loci, boosting sensitivity for targeted contacts. HiChIP [104] and Proximity Ligation-Assisted ChIP-seq (PLAC-seq) [105] integrate Hi-C with ChIP-seq to couple chromatin interactions with histone modifications or protein binding, improving signal-to-noise around regulatory factors while introducing antibody/peak dependence. Micro-C [106] replaces restriction digestion with MNase to achieve nucleosome-scale resolution, sharpening loops and boundaries while remaining a fixed, ligation-based snapshot. Region Capture Micro-C (RCMC) extends Micro-C with dense tiling capture, enabling ultra-deep, nucleosome-resolution contact maps over selected megabase-scale regions. By focusing sequencing on targeted loci, RCMC provides unprecedented detail of chromatin folding and reveals fine-scale structures such as nested microcompartments. The method can be readily integrated with complementary assays such as CUT&RUN and Assay for Transposase-Accessible Chromatin with high-throughput sequencing (ATAC-seq) and applied in perturbation settings, thereby linking 3D structural rewiring to local chromatin features [107]. In addition, multi-contact methods such as MC-4C [108], Tri-C [109], Nano-C [50], and Pore-C [110] can capture higher order interactions involving more than two loci. Complementing these 3C-derived assays, non-3C strategies such as SPRITE (Split-Pool Recognition of Interactions by Tag Extension) [111] and GAM (Genome Architecture Mapping) [112] provide orthogonal means to map nuclear contacts without relying on proximity ligation. These advances make it possible to trace the physical interactions between distant genomic loci, clarifying the 3D structure of the genome and offering information on the location of chromatin insulators in relation to enhancers, promoters, and other developmental components.

The development of genome editing technologies, such as CRISPR-Cas9, has allowed for precise genome manipulation, revolutionizing the area of chromatin biology [113]. Researchers can modify the function of insulator proteins, create mutations in chromatin insulator sequences, and add or remove insulator elements at certain genomic loci using CRISPR-based methods. A powerful technique for studying chromatin insulators at the cellular level is single-cell genomics [114,115]. Through the analysis of individual cell chromatin accessibility, histone modifications, and transcriptional activity, researchers can examine the regulatory landscapes and chromatin states that vary throughout distinct cell types.

To understand the mechanisms of gene regulation, it is essential to develop novel methods for imaging so that we can capture chromatin at all stages of transcription. A recent study done in zebrafish made it possible to analyze chromatin at the individual nucleosome level. This technique, called chromatin expansion microscopy, or chromExM, demonstrated that PolII and the TF did not concurrently occupy the same areas throughout the entire nucleus. Instead, chromatin changed its configuration based on the constituents that were present in different combinations [116]. Hi-M is a sequential imaging method that uses multiplexing to simultaneously measure chromatin organization and transcription in single cells [117]. The use of Hi-M in whole *Drosophila* embryos demonstrated that transcriptional states of different cell types are not controlled by the vicinity of distant cis-regulatory elements and promoters [118]. Optical reconstruction of chromatin architecture (ORCA) has been used at the *Drosophila* hox cluster to identify Polycomb-dependent and -independent borders [80]. Sequential fluorescence *in situ* hybridization (seq-FISH+) is an advanced multiplexed, sequential imaging technique that has facilitated the simultaneous profiling of genome structure along with a wide array of epigenetic features and transcripts [119,120]. MERFISH (multiplexed error-robust fluorescence in situ hybridization) is another technique that is used to visualize chromatin in the context of transcription [121]. A recent review describes most of these techniques in detail [122].

## Discussion

In this review, we discussed insulator elements and their role in transcription regulation both in mammals and in *Drosophila*. Insulators play an important role in genome packaging, and this packaging has functional consequences. A major functional role of insulator elements is in the insulation of TADs. Interaction between genes and regulatory elements is favored intra-TAD compared to inter-TAD. However, it is important to remember that there is a great deal of cell-to-cell variation within a population and that TADs and chromatin loops are dynamic structures rather than fixed structures. According to recent research, the auxiliary factors and redundancy of insulator proteins work together to ensure that the loss of any one protein has comparatively minor consequences on gene expression. It is challenging to disentangle transcription from the regulatory mechanisms that govern it, such as chromatin insulators, yet comprehending the role that transcription plays in compartmentalization and 3D genomic architecture is a crucial problem to solve. Future research should focus on integrating novel imaging and genome-editing tools to uncover the interplay between transcriptional regulation and 3D chromatin architecture. Next-generation single-cell and multi-omics assays can close this gap by directly linking 3D architecture to gene output. Pairing single-cell contact maps with joint chromatin-state and transcriptome profiling will quantify cell-to-cell variability in boundary strength/TAD structure and its transcriptional consequences. We propose live-cell imaging of labeled insulators and regulatory loci to quantify dynamic changes in TADs. This should measure boundary mobility and loop lifetimes across cell states and throughout the cell cycle. We also propose single-molecule studies to define how insulator proteins modulate chromatin loop formation. Understanding these mechanisms will enhance our knowledge of gene regulation and its implications for development and disease.

**Perspectives**

- The function of chromatin insulators is very important in genome organization and transcription regulation. Insulators define chromatin domains and mediate interactions between regulatory elements, which play critical roles in development, differentiation, and disease and provide insights into fundamental processes and possible therapeutic targets.
- Insulators, such as CCCTC-binding factor in mammals and boundary element-associated factor in *Drosophila*, act as key mediators of enhancer blocking, barrier activity, and chromatin looping. There is redundancy and diversity of insulator proteins in different species. Recent technological advances using Hi-C and CRISPR have pointed out the role of insulators in topologically associating domains and three-dimensional (3D) genome architecture.
- The interplay between transcriptional regulation and 3D chromatin architecture needs to be explored. Bringing together recent advances in approaches such as ChromEM tomography (ChromEMT) and single-cell genomics could elucidate the mechanisms underlying chromatin boundaries and their roles in health and disease.

## Open Access

This article has been published open access under our Subscribe to Open programme, made possible through the support of our subscribing institutions, learn more here: https://portlandpress.com/pages/open_access_options_and_prices#conditional

## Abbreviations

Abd-B: Abdominal-B
BEAF: boundary element-associated factor
3C: chromosome conformation capture
CP190: centrosomal protein 190
CTCF: CCCTC-binding factor
CdLS: Cornelia de Lange syndrome
ChromEMT: ChromEM tomography
DREF: DNA replication-related element-binding factor
E–P: enhancer–promoter
Fab-7: Frontabdominal-7
Fab-8: Frontabdominal-8
GAF: GAGA-associated Ffactor
IBP: insulator binding protein
IDH: isocitrate dehydrogenase
LCR: locus control region
MAZ: Myc-associated zinc finger protein
NIPBL: Nipped-B-like
ORCA: Optical reconstruction of chromatin architecture
PDGFRA: platelet-derived growth factor receptor alpha
PLAC-seq: Proximity Ligation-Assisted ChIP-seq
PRE: Polycomb response element
RCMC: Region Capture Micro-C
SEs: super-enhancers
TADs: topologically associating domains
TEs: tethering elements
WAPL: Wings apart-like protein homolog
Zw5: Zeste-white 5
scs: specialized chromatin structure
